# Handgrip Strength and Muscle Quality: Results from the National Health and Nutrition Examination Survey Database

**DOI:** 10.3390/jcm12093184

**Published:** 2023-04-28

**Authors:** Zhangxin Wen, Jiaxuan Gu, Rong Chen, Qinyi Wang, Na Ding, Lingqiong Meng, Xiangbing Wang, Hong Liu, Zhifeng Sheng, Houfeng Zheng

**Affiliations:** 1 Department of Metabolism and Endocrinology, Zhuzhou Hospital of Xiangya School of Medicine, Central South University, 116 Changjiang South Road, Zhuzhou 412007, China; 2 Diseases & Population (DaP) Geninfo Lab, School of Lifesciences, Westlake University, 600 Dunyu Road, Hangzhou 310030, China; 3 Westlake Laboratory of Life Sciences and Biomedicine, 18 Shilongshan Street, Hangzhou 310024, China; 4 Key Laboratory of Endocrinology, Department of Endocrinology, National Health and Family Planning Commission, Peking Union Medical College Hospital, Chinese Academy of Medical Sciences, Peking Union Medical College, Shuaifuyuan No. 1, Beijing 100730, China; 5 National Clinical Research Center for Metabolic Diseases, Hunan Provincial Key Laboratory of Metabolic Bone Diseases, Department of Metabolism and Endocrinology, Health Management Center, The Second Xiangya Hospital of Central South University, 139 Middle Renmin Road, Changsha 410011, China; 6 Department of Nutritional Sciences, Rutgers University, New Brunswick, NJ 08901, USA; 7 Divisions of Endocrinology, Metabolism and Nutrition, Rutgers-Robert Wood Johnson Medical School, New Brunswick, NJ 08901, USA

**Keywords:** sarcopenia, handgrip strength, appendicular lean mass index, age, physical activity

## Abstract

Background: Handgrip strength (HGS) and the appendicular lean mass index (ALMI) are important determinants of sarcopenia. Muscle quality (MQ) is a measure of muscle strength relative to muscle mass. We examined trends in handgrip strength, the appendicular lean mass index, and analyzed their relationship with age, anthropometry, and body composition in a sample of participants in the United States (US). Methods: This cross-sectional study analyzed data from 14,741 US males (49.7%) and females (50.3%) 6–80 years old who responded to the National Health and Nutrition Examination Survey (NHANES) from 2011 to 2014. Dual X-ray absorptiometry was used to measure appendicular skeletal muscle mass. HGS was evaluated using the Takei Digital Grip Strength Dynamometer. Smoothed normative curves for HGS and the ALMI were constructed using a generalized additive model. Multiple regression analyses were used to examine associations of HGS and the ALMI with age, nutrition-related factors, physical activity, and body composition. Results: Mean HGS and the ALMI declined with advancing age. While mean HGS increased with the ALMI, it decreased with the fat mass index. HGS increased in males with an increase in body mass index, energy intake, the ALMI, and vitamins; however, HGS in females increased with albumin, but it had a negative association with the fat mass index and age, but not with increasing adiposity. Conclusions: HGS and the ALMI change with age: HGS increases with age, then stabilizes and declines; the ALMI increases with age, then stabilizes. In addition, we provide evidence for the effect of anthropometry, nutrition, physical activity, and body composition on HGS and the ALMI in US population.

## 1. Introduction

Depending on its severity, sarcopenia can increase the incidence of falls and bone fractures, adding to the risk of mobility disability and loss of self-care ability among older adults [1]. The term “sarcopenia” was first used to describe the age-associated decline in skeletal muscle mass. Over the course of updates to the guidelines for sarcopenia (2010–2019), the field has moved from focusing primarily on muscle mass to integrating muscle strength and muscle mass as part of a more comprehensive definition of sarcopenia [2,3].

Computed tomography and magnetic resonance imaging are considered the gold standards for the quantification of skeletal muscle mass, but they are time-consuming and expensive [4]. Dual-energy X-ray absorptiometry (DXA) is recommended for clinical implementation due to its high reliability and low cost [5]. Appendicular lean soft tissue mass, as measured with the DXA, is strongly correlated with the total-body skeletal muscle mass, as quantified with magnetic resonance imaging [6]. Lean soft tissue, as measured with the DXA, is associated with sarcopenia and adverse health status [7,8]. MQ expresses muscle strength relative to muscle mass. Measures of appendicular (the sum of arms and legs) lean mass (ALM) parameters (ALM, ALM/h^2^) are preferred over the total lean mass, as the latter is subject to more confounding of organ masses in the trunk region [9]. The ALM parameters are also more relevant to activities of daily living and are recommended in definitions of sarcopenia [2].

Low handgrip strength (HGS), appendicular lean mass index (ALMI), and physical performance are components of a diagnosis of sarcopenia [2]. The measurement of HGS is simple and inexpensive, and HGS correlates strongly with measurements of muscle strength from other muscle groups, including the lower limbs, thereby serving as a reliable surrogate for complicated measurements [10]. Assessment of HGS is advised for routine use in hospital practice, clinical specialties, and community health settings [10].

Sarcopenia has long been associated with aging and older adults, but the development of sarcopenia is known to begin earlier in life [11]. Evidence supporting sarcopenia-related sex differences and factors associated with the effects of sarcopenia is limited; it is needed to facilitate the appraisal of muscle strength and to identify individuals with or without risk factors for sarcopenia.

Using the 2011–2014 National Health Examination Survey data, this study aims to explore the trend of grip strength and the ALMI with age, and to calculate two cut-off values using threshold-effect analysis. The second purpose is to discover the effects of anthropometry, nutrition status (Energy (kcal) and albumin level), physical activity, and body composition on HGS and muscle quality.

## 2. Methods

This cross-sectional study was conducted in accordance with the guidelines of the Strengthening the Reporting of Observational Studies in Epidemiology (STROBE) statement [12].

### 2.1. Data Source and Study Population

The NHANES is a cross-sectional, nationwide survey designed to assess the health and nutritional status of children and adults in the US; it has been conducted periodically since the 1960s. The study population, which is considered a representative sample of the US population, was recruited using a multistage, stratified sampling method. Further details of the survey’s methodology can be found elsewhere [13].

The 2011−2014 survey consists of data released in 2-year cycles: 2011−2012 (*n* = 9756) and 2013−2014 (*n* = 10,175). Data from these two cycles were combined (*n* = 19,931), and adults ≤ 80 years of age who underwent a DXA or HGS assessment were selected (*n* = 14,741) for the analysis. The final sample of 14,741 older adults was analyzed during the winter of 2021.

The National Center for Health Statistics Ethics Review Board approved the protocols for the NHANES, and all the participants provided written informed consent.

### 2.2. Clinical and Laboratory Evaluations

Details of the data-collection procedures have been reported elsewhere [13]. Information about participants’ demographics, health and medical history, and prescribed medications were obtained during home interviews. Trained personnel in a mobile examination center performed all other procedures.

The NHANES data included the participants anthropometric measurements, information about their lifestyles and physical activity, and laboratory test results. We categorized race/ethnicity as non-Hispanic white, non-Hispanic black, Hispanic (Mexican American, other Hispanic), or other. We defined smokers as individuals who were current smokers, never smoked, or ever smoked. Participants who had smoked at least 100 cigarettes in life and now smoked cigarettes every day or sometimes were categorized as current smokers; those who had smoked at least 100 cigarettes in life and now discontinued smoking were classified as ever smokers; those who had never smoked at least 100 cigarettes in life were classified as never smokers [14].

We assessed the participants’ amount and frequency of alcohol consumption based on a self-report questionnaire. A drink indicates a 12 oz. beer, a 5 oz. glass of wine, or 1.5 oz. liquor. Drinking status was ascertained if participants had at least 12 alcohol drinks/year [15]. All details of study variables and covariates in the present study could be accessed through the webpage www.cdc.gov/nchs/nhanes (accessed on 29 May 2022).

### 2.3. Handgrip Strength (kg)

Grip strength measurements were taken in the 2011–2014 survey, following the NHANES 2011–2014 protocol. In accordance with the Muscle Function Procedures Manual, grip strength was evaluated using the Takei Digital Grip Strength Dynamometer, Model T.K.K.5401 (Takei Scientific Instruments Co., Niigata, Japan). Participants aged 6 years and older, who did not meet any of the exclusion criteria, were eligible for grip strength measurements. Prior to the measurement of handgrip strength, the dynamometer was adjusted to the size of each hand of the participants, who were in a standing position with their arm straight down and wrist in a neutral position. They were asked to squeeze the dynamometer as hard as possible using one hand. The test was repeated three times on alternating hands with a rest period of 60 s between measurements of the same hand. The NHANES reported a combined HGS as the sum of the largest reading from each hand, which was expressed as kg. The mean-combined HGS achieved in all 6 trials was used in the analysis [16], as defined in the Sarcopenia Definitions and Position Statements of the Sarcopenia Definition and Outcomes Consortium [17].

### 2.4. Body Mass Components Dataset

Body composition was assessed using the DXA, which was calibrated daily, and all results were obtained using Hologic QDR-4500 software, version Apex 3.2. (Hologic, Bedford, MA, USA). Body fat mass (kg and %), total lean soft tissue (kg), arm appendicular skeletal muscle (ASM) mass (kg), and leg ASM mass (kg) were measured in the eligible participants.

The ALM, a well-recognized proxy for skeletal muscle mass [14], was calculated by summing the lean mass (excluding bone mineral content) of the right and left legs and right and left arms, as measured by the DXA. The ALMIs of the participants were calculated using ALM/height^2^; the age ranges of all the body mass components and variables in the dataset (including body mass and height) were limited to 8–59 years because participants older than 59 years were not eligible for the DXA measurements. Additional details regarding the DXA procedures are available on the NHANES website [18].

### 2.5. Covariates

The demographic and lifestyle factors used as covariates were as follows: age (years); sex (male, female); serum 25-hydroxy vitamin D (25(OH)D); caloric intake (energy intake); and information about physical activity was based on participants’ responses to the Global Physical Activity Questionnaire. Liquid chromatography–tandem mass spectrometry was used to measure the serum 25(OH)D [19]. The DcX800 method was used to measure the albumin concentration as a bichromatic digital endpoint method. The automated multiple-pass method was used to determine total energy intake (kilocalories per day) by using the interviewer-administered 24 h dietary recall on that day [20,21]. Survey participants aged ≥12 years completed the dietary interview on their own, proxy-assisted interviews were conducted with children aged 6–11 years. This questionnaire assessed three types of physical activity: leisure time, occupation-related, and transportation-related [22]. Each type of physical activity included questions to assess the intensity (vigorous or moderate), frequency (per week), and duration (minutes) of physical activity in a typical week. Subjective estimates of exercise intensity were determined using activity codes and the metabolic equivalent of the task (MET) for all physical activities performed over the last seven days. These data were then used to determine the weighted MET-minutes per week (MET min/week) for walking and for moderate and vigorous activity [23,24].

### 2.6. Statistical Analysis

Due to the complexity of the NHANES’ design, we selected appropriate sample weights, stratification, and clustering to ensure a sample representative of the US population [25].

Participants’ characteristics and descriptive data on all outcomes are presented as mean ± SD or frequencies (percentages). Regression modelling revealed a non-linear (quadratic) association of age with HGS and the ALMI. Spline curves were fitted and compared with the quadratic regression plots to model the goodness of fit measure for the adequacy of the quadratic model. Multi-variable models were developed to identify the factors most associated with HGS and the ALMI, including age, anthropometry, measures of body composition, nutritional status, and physical activity in the analysis.

All analyses were conducted separately for males and females. Modeling was performed with the statistical software R version 4.2. (R Foundation for Statistical Computing, Vienna, Austria, http://www.R-project.org) and EmpowerStats (http://www.empowerstats.com, X&Y Solutions, Inc., Boston, MA, USA). Statistical significance was set at *p* < 0.05 (two-sided).

## 3. Results

### 3.1. Selected Participants

**Figure 1:**
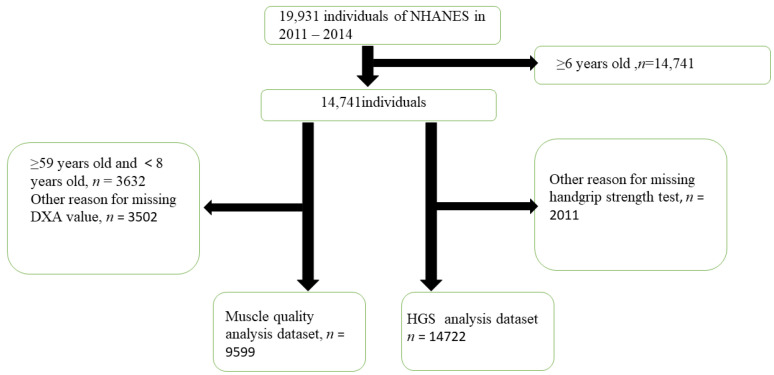
Flowchart of population included in our final analysis, NHANES, USA, 2011–2014.

Data from 14,741 males and females were included in the present study based on strict inclusion and exclusion criteria (Figure 1). We took the HGS values of the dominant hands, and according to the results of our Table S1, there was no statistical difference between the HGS of the male and female dominant hands and the grip strength of the non-dominant hands.

#### 3.1.1. Baseline Characteristics

Among the 14,722 participants with measurements of HGS, 9599 also had the ALMI data. Table 1 presents the participants’ characteristics with measurements of HGS. The mean weight, height, waist circumference, albumin, and energy intake were higher among males than females. Similarly, physical activity levels were higher among males (all *p* values < 0.001). Table S2 presents the participants’ characteristics who underwent the ALMI; this result is consistent with trends in the Table 1. Missing measurements of other indicators included 452 measurements of height, 668 measurements of weight, and 415 measurements of waist circumference. Dummy variables were used to indicate missing covariates.

#### 3.1.2. Curve Fitting and Threshold-Effect Analysis

**Figure 2:**
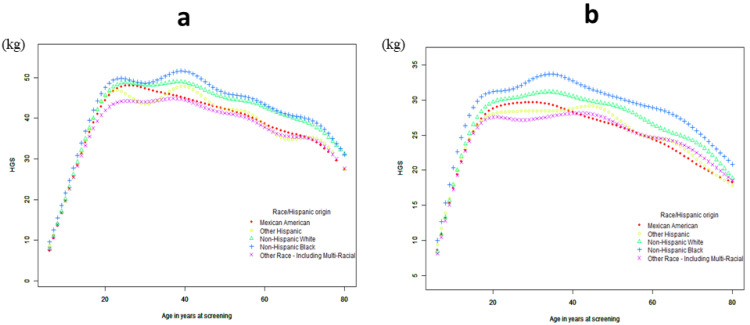
The association between age and HGS for male (**a**) and female (**b**). A generalized additive model was used to test the relationship of age with HGS.

**Figure 3:**
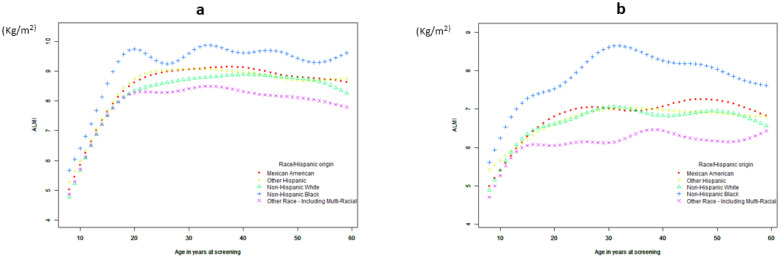
The association between age and ALMI for male (**a**) and female (**b**). A generalized additive model was used to test the relationship of age with ALMI.

A generalized additive model was used to test the relationship of age with HGS and the ALMI. Figure 2 shows changes in HGS with age. At first, HGS increased significantly, showing a piecewise linear relationship with age. Based on the results of the threshold-effect analysis (Table 2 and Table S3), we identified two cut-off points: 18 was the first inflection point for women, and 20 for men. When a two-piecewise logistic regression model was used to evaluate the threshold-effect of the fitted curve, the log-likelihood ratio test of age at the inflection point of 18 was statistically significant (*p* = 0.005), suggesting that the two-piecewise regression model was appropriate for examining the relationship between age and HGS (Table 2 and Table S3). When age <18 years, HGS increased with age; when age ≥ 18 years, the HGS of females did not change significantly with age; and when age >72 years in males (Table S5) and age >66 years in females (Table S6), the HGS of females decreased significantly. Figure 3 shows changes in the ALMI with age. Further analyses indicated there was only one inflection point. As shown in Figure 3 and Table 3 and Table S4, the ALMI increased as age increased, reaching approximately 19 in females and 21 in males, and then remained stable.

#### 3.1.3. Handgrip Strength and Body Composition

A non-linear age-related decline in HGS (Figure 2; Table 4) was found. Results of the multiple logistic regression analysis (Table 4) indicated a significant association of HGS with age, body mass index (BMI), and energy intake among males and females, and HGS was associated with vitamin D in males and albumin in females. A separate model revealed a positive association between HGS and the ALMI in males (β = 3.006, *p* < 0.001) and females (β = 2.71, *p* < 0.001), and HGS was negatively associated with the fat mass index (FMI) in males (β = −0.593, *p* < 0.001) and females (β = −0.516, *p* < 0.001). These associations were sustained after adjustments were made for smoking, drinking, and physical activity, but HGS has no significant correlation with physical activity, as shown in Models 1 and 2.

A non-linear age-related decline in the ALMI (Figure 3; Table 5) was also observed, and the ALMI was associated with age, BMI, albumin, vitamin D, and energy intake in both males and females. Furthermore, the ALMI was negatively associated with metabolic syndrome (MetS) in women after adjustments for age, BMI, ethnicity, albumin, vitamin D, energy intake, drinking, smoking, and physical activity. There is no significant correlation with physical activity, but there is a correlation between the ALMI and physical activity in women.

## 4. Discussion

In the present study, we observed that HGS and the ALMI vary with age, anthropometry, nutrition, physical activity, and measures of body composition in adults from the US, and we reported sex-specific patterns of correlations with sarcopenia.

The mean HGS and ALMI were higher in males than in females across all age groups. The specific profile of body composition and other confounders by sex might explain why the results were not comparable between males and females. Our data are consistent with the observations of healthy non-obese participants between 18 and 94 years old [26].

The precise age of the individual at the onset of sarcopenia is subject to debate [27]. We found that HGS peaks during early adulthood and declines with increasing age in both males and females. We also found that the patterns of HGS changes are different between males and females, and that the HGS of females peaked at 18–66 years old. This finding is inconsistent with the normative data from the UK, where an analysis of combined data from 12 studies reported that the HGS of females peaked around the fourth decade of life (26–42 years old) [28]. We found that HGS remained essentially stable during the fourth and seventh decades of life, followed by a subsequent decrease with advancing age. We also found that the second inflection point was 66 for females and 72 for males, with the HGS of females declining earlier than that of males. This finding is consistent with that of a study that examined the muscle strength and age of Australian participants [29], and a study of the general population, in which the age-related decline in HGS ranged from 20 to 50 years of age [30]. The decline increased after 50 years of age, suggesting a change point in HGS at approximately 50 years old [24]. The rapid decline in HGS after age 50 is likely caused by declining estrogen levels that accompany menopause [31]. A large-scale study of Caucasians showed a more rapid gain in strength with a peak at approximately age 24, while in females, a peak is reached at around 29 years old. The average HGS remains stable up to age 40–44 and declines thereafter in both males and females [32]. The inconsistencies between their data and our data may be due to different recruitment strategies and inclusion/exclusion criteria. Individuals with arm or hand pain and/or arthritis lasting more than one month were excluded from that study and the population was ≥ 18 years old.

The ALMI is a good indicator of skeletal muscle mass, and it is commonly used to define sarcopenia [33]. There have been a few reports about the ALMI in the research literature [34]; a study of Caucasians [35] reported a decreasing trend in the mean ALMI with increasing age in both males and females, which was not consistent with our results because we included a population with a mean age less than 60 years.

There are a few studies on the factors influencing muscle strength and quality [27,28,29,36]. Previous research on this topic has used relatively small samples and participants with a narrower age range, but these studies did not adjust for important covariates (e.g., physical activity level, vitamin D, energy intake, ethnicity, smoking status, and alcohol intake). To our knowledge, the present study is the first one in which demographic characteristics, nutritional factors, and physical activity have been included in the analysis.

Previous studies show that males and females with malnutrition have significantly lower HGS [36]. One of the most important effects of malnutrition is insufficient energy intake. In this study, multiple regression analysis revealed a significant positive correlation of HGS with energy intake, and albumin level was an indicator of nutritional status. The results of our multivariate data analysis revealed a significant relationship between HGS-related factors, including aging, and the level of serum albumin in females. Previous studies have shown an association of hypoalbuminemia [37] with HGS and the ALMI in females. Ensuring adequate energy intake and preventing malnutrition are very important for muscle strength. However, a study conducted in six US medical centers reported a significant relationship of serum albumin with ASM mass and HGS in men [38], but low serum albumin within the normal range was not a risk factor for loss of HGS in older men, which is inconsistent with our findings. A possible reason is that the population included in this study consisted of men over 65 years old with a higher incidence of hypoalbuminemia. Sex differences between correlations of muscle-related parameters in the current study might be due to differences in the associations of each factor: females had a lower albumin level and sex differences were found in the distribution of lean mass, with males tending to have larger amounts of upper body lean mass [39].

The finding that HGS is associated with a higher BMI is consistent with prior observational and prospective studies [40,41], which is consistent with the results of our study. Similar associations were not observed among females in a study by the HALCyon program, which is consistent with our findings of weaker associations among females than males [41]; however, BMI does not indicate body shape or health endpoints. We examined the association of the ALMI and FMI with HGS, and found that HGS decreased with an increasing FMI, which concurs with previous observations [29].

Our study found that the ALMI had a significant positive relationship with vitamin D, which is consistent with findings that vitamin D deficiency is associated with poor muscle mass and impaired physical performance in older adults [42]. Previous studies have also reported that 25 (OH)D might be important for ALMI [43]. The vitamin D receptor is expressed in muscle tissue [44], and its activation results in de novo muscle protein synthesis, as confirmed by a randomized placebo-controlled clinical trial with postmenopausal women [45]. Supplementation of vitamin D may be a simple, safe, and cost-effective strategy to support a healthier body composition of an older adult. Our finding that the ALMI is positively associated with physical activity might be the reason why the sensitivity of skeletal muscle tissue to anabolic stimuli, such as physical activity or protein intake, might be reduced in sedentary older adults [46]—so, physical inactivity causes a decline in muscle strength.

Our study has several strengths and limitations. First, a major strength is that compared to other studies, we have included more factors related to sarcopenia, such as physical activity level, vitamin D, energy intake, ethnicity, smoking status, and alcohol intake, which can help evaluate the factors influencing sarcopenia in a more comprehensive manner. Second, objective measures of lean mass and HGS were obtained according to standard protocols. Although participants were encouraged to demonstrate maximal HGS, we cannot exclude the possibility of a suboptimal effort. Third, the use of the DXA for measures of body composition is the gold standard. Fourth, a generalized additive model was used in this study to clarify the non-linear relationship between age and HGS. Fifth, we assessed the study population using a random sampling technique and covered all the stages of life, so the data were not analyzed by disease. Finally, for the first time, we calculated two cut-off values using threshold-effect analysis to get the turning point more accurately.

Our study also has several limitations. First, the body composition data were from people under the age of 60 years, so the body composition distribution and factors influencing the older adults could not be analyzed in this study. Second, the study’s cross-sectional design does not permit us to infer causal relationships. Finally, the 2011–2014 NHANES database might not provide an accurate reflection of current population dynamics.

## 5. Conclusions

In conclusion, the overall pattern of decline in HGS and the ALMI with advancing age was non-linear. In a relatively representative American group, we examined the influence of anthropometry, nutrition, physical activity, and body composition on the ALMI and HGS. These findings might be useful for risk estimation and evaluation of interventions for sarcopenia.

## Figures and Tables

**Table 1:** Population-weighted anthropometry and demographics and behavioral characteristics of the individuals who performed the HGS.

	HGS		
	Total	Male	Female
Anthropometry and demographics			
Age (year)	39.85 ± 0.43	39.04 ± 0.50	40.64 ± 0.43 *
Race/Hispanic origin (%)			
Mexican American	9.58	8.75	7.39 *
Other Hispanic	6.12	5.79	5.83
Non-Hispanic White	64.30	66.98	67.15
Non-Hispanic Black	12.05	10.66	12.10 *
Other Race—Including Multi-Racial	7.95	7.82	7.54
Weight (kg)	76.42 ± 0.36	81.48 ± 0.44	71.51 ± 0.52 *
Smoking (%)			
Never	41.9	40.35	43.82
Yes	53.44	54.16	52.56
Ever	4.66	5.49	3.62 *
Drinking (%)			
Yes	42.95	46.6	39.2 *
No	57.05	53.4	60.8 *
Waist Circumference (cm)	94.03 ± 0.33	95.63 ± 0.39	92.46 ± 0.43 *
Height (cm)	165.37 ± 0.21	171.31 ± 0.28	159.60 ± 0.22 *
BMI (kg/m^2^)	27.42 ± 0.14	27.12 ± 0.13	27.71 ± 0.19 *
Physical activity (Mets score)	3299.16 ± 171.23	4395.82 ± 252.01	2238.04 ± 169.05 *
Vitamin D (nmol/L)	69.51 ± 0.15	66.66 ± 1.00	72.28 ± 1.22 *
Albumin (g/l)	43.15 ± 0.06	44.14 ± 0.08	42.20 ± 0.07 *
Energy (kcal)	2166.14 ± 10.72	2489.55 ± 14.36	1850.86 ± 11.14 *

* *p* < 0.05.

**Table 2:** Threshold-effect analyses of association between age and HGS for men.

HGS	β(95%CI)	Detail−*p*
Mexican American		
<20	2.89 (2.74, 3.04)	
>20	−0.24 (−0.28, −0.20) <0.0001	−3.14 (−3.28, −2.99) <0.0001
Other Hispanic		
<20	2.82 (2.66, 2.98) <0.0001	
>20	−0.22 (−0.26, −0.18) <0.0001	−3.04 (−3.22, −2.86) <0.0001
Non−Hispanic White		
<20	3.11 (3.01, 3.21) <0.0001	
>20	−0.22 (−0.24, −0.20) <0.0001	−3.33 (−3.44, −3.22) <0.0001
Non−Hispanic Black		
<20	3.01 (2.89, 3.12) <0.0001	
>20	−0.21 (−0.24, −0.18) <0.0001	−3.22 (−3.35, −3.09) <0.0001
Other Race—Including Multi-Racial		
<20	2.74 (2.61, 2.87) <0.0001	
>20	−0.21 (−0.24, −0.18) <0.0001	−2.94 (−3.09, −2.80) <0.0001
Total		
<20	3.01 (2.95, 3.06) <0.0001	
>20	−0.22 (−0.23, −0.21) <0.0001	−3.22 (−3.29, −3.16) <0.0001

**Table 3:** Threshold-effect analyses of association between age and the ALMI for men.

ALMI	β (95%CI)	Detail−*p*
Mexican American		
<21	0.3 (0.3, 0.3) <0.00	
>21	−0.0 (−0.0, −0.0) 0.008	−0.3 (−0.3, −0.3) <0.001
Other Hispanic		
<21	0.3 (0.3, 0.3) <0.001	
>21	−0.0 (−0.0, −0.0) 0.004	−0.3 (−0.3, −0.3) <0.001
Non−Hispanic White		
<21	0.3 (0.3, 0.3) <0.001	
>21	−0.0 (−0.0, 0.0) 0.146	−0.3 (−0.3, −0.3) <0.001
Non−Hispanic Black		
<21	0.3 (0.3, 0.3) <0.001	
>21	−0.0 (−0.0, −0.0) <0.001	−0.3 (−0.4, −0.3) <0.001
Other Race—Including Multi-Racial		
<21	0.3 (0.2, 0.3) <0.001	
>21	−0.0 (−0.0, −0.0) <0.001	−0.3 (−0.3, −0.2) <0.001
Total		
<21	0.3 (0.3, 0.3) <0.001	
>21	−0.0 (−0.0, −0.0) <0.001	−0.3 (−0.3, −0.3) <0.001

**Table 4:** Models for predicting HGS (population aged over 20 years old).

Category Variables		Model		Coefficient (B)	*p* Value	R2 Adjusted
HGS	Male	1				−180.7177
			Age	−0.22	<0.0001	
			BMI	0.28	<0.0001	
			Energy	0.00057	0.0004	
			Vitamin D	0.0315	<0.0001	
			Constant	42.868	<0.0001	
	Female	1	age	−0.15855	<0.0001	−173.6247
			BMI	0.13017	<0.0001	
			Energy	0.0003	0.0059	
			Albumin	0.04352	<0.0001	
			Constant	30.1887	<0.0001	
	Male	2	Age	−0.06726	0.00167	−112.4302
			Vitamin D	0.0236	0.00916	
			FMI	−0.593	<0.0001	
			ALMI	3.00662	<0.0001	
			Constant	24.4784	<0.0001	
	Female	2	Age	−0.01874	<0.0001	−99.7666
			Albumin	0.04517	0.0005	
			FMI	−0.5168	<0.0001	
			ALMI	2.71318	<0.0001	
			Constant		<0.0001	

Model 1: Age, BMI, Ethic, Albumin, Vitamin D, Energy, Drink, Smoke, Physical activity. Model 2: Age, Albumin, Vitamin D, Energy, Drink, Smoke, Physical activity, FMI, ALMI.

**Table 5:** Models for predicting the ALMI (population aged >20 to >20 plus <59).

Category Variables		Model		Coefficient (B)	*p* Value	R2 Adjusted
ALMI	Male	1				−36.6993
			Age	−0.01173	0.000034	
			BMI	0.18884	<0.000001	
			Albumin	0.05302	<0.000001	
			Energy	0.00034	<0.000001	
			Vitamin D	0.00277	0.005891	
			Constant	0.02210	0.956095	
	Female	1	Age	−0.01173	0.000034	−13.9582
			BMI	0.18675	<0.0001	
			Albumin	0.01003	0.023860	
			Vitamin D	0.00255	0.000473	
			Energy	0.00009	0.000007	
			Physical activity	0.00001	0.041227	
			Constant	2.02312	<0.0001	

Model 1: Age, BMI, Ethic, Albumin, Vitamin D, Energy, Drink, Smoke, Physical activity.

## Data Availability

The datasets used and/or analyzed during the current study are available from the corresponding author on reasonable request.

## Supplementary Materials

The following supporting information can be downloaded at: https://www.mdpi.com/article/10.3390/jcm12093184/s1, Table S1: Comparison of handgrip strength between dominant and nondominant hands. Table S2: Population-weighted Anthropometry and demographics and behavioral characteristics of the individuals who performed the ALMI. Table S3: Threshold effect analyses of association between age and HGS for women. Table S4: Threshold effect analyses of association between age and ALMI for women. Table S5: Threshold effect analyses of association between age and HGS for men. Table S6: Threshold effect analyses of association between age and HGS for women.
