# Supplementary material for: Handgrip Strength and Muscle Quality: Results from the National Health and Nutrition Examination Survey Database
Source: J Clin Med. 2023 Apr 28;12(9):3184. doi: 10.3390/jcm12093184 (PMC10179381; doi:10.3390/jcm12093184)
Supplement: Supplementary file 1 [file jcm-12-03184-s001.zip › jcm-2148661-supplementary.pdf]

Table S1. Comparison of handgrip strength between dominant and nondominant hands.

	Dominant hand	Non-dominant hand	P value
Male	40.85 ± 0.20	40.37 ± 0.39	0.4103
Female	26.84 ± 0.14	26.52 ± 0.27	0.3267

Table S2. Population-weighted Anthropometry and demographics and behavioral characteristics of the individuals who performed the ALMI.

	ALMI	
	Male	Female
Anthropometry and demographics		
Age (year)	32.9 ± 0.4	33.2 ± 0.5*
Race/Hispanic origin (%)		
Mexican American	11.8	10.5*
Other Hispanic	6.6	6.9
Non-Hispanic White	61.4	61.2
Non-Hispanic Black	11.6	12.9
Other Race - Including Multi-Racial	8.6	8.5
Weight (kg)	81.0 ± 0.5	71.1 ± 0.6 *
Smoking (%)		
Never	48.3	52.7
Yes	45.0	43.6
Ever	6.7	3.7
Drinking (%)		
yes	47.4	41.1
no	52.6	58.9
Waist circumference (cm)	94.1 ± 0.4	90.8 ± 0.5 *
Height (cm)	172.1 ± 0.3	160.8 ± 0.2 *
BMI (kg/m$^{2)}$	26.9 ± 0.2	27.2 ± 0.2 *
Physical activity（Mets score）	4670.6 ± 188.8	2594.8 ± 256.0 *
Vitamin D(nmol/L)	63.6 ± 1.1	67.9 ± 1.4 *
Albumin(g/l)	44.7 ± 0.1	42.6 ± 0.1 *
Energy (kcal)	2566.4 ± 19.9	1895.6 ± 14.9 *

* $p < 0.05$.

Table S3. Threshold effect analyses of association between age and HGS for women.

HGS	β（95%CI）	Detail-P
Mexican American		
<18	1.74 (1.65, 1.83) <0.0001	
>18	−0.15 (−0.17, −0.13) <0.0001	−1.89 (−1.99, −1.79) <0.0001
Other Hispanic		
<18	1.63 (1.50, 1.76) <0.0001	
>18	−0.14 (−0.16, −0.11) <0.0001	−1.77 (−1.91, −1.63) <0.0001
Non-Hispanic White		
<18	1.96 (1.88, 2.05) <0.0001	
>18	−0.16 (−0.17, −0.15) <0.0001	−1.98 (−2.08, −1.89) <0.0001
Non-Hispanic Black		
<18	1.84 (1.74, 1.94) <0.0001	
>18	−0.14 (−0.15, −0.12) <0.0001	−1.97 (−2.08, −1.86) <0.0001
Other Race - Including Multi-Racial		
<18	1.66 (1.55, 1.77) <0.0001	
>18	−0.12 (−0.14, −0.11) <0.0001	−1.78 (−1.90, −1.66) <0.0001
Total		
<18	1.86 (1.81, 1.91) <0.0001	
>18	−0.15 (−0.16, −0.15) <0.0001	−2.01 (−2.06, −1.96) <0.0001

Table S4. Threshold effect analyses of association between age and ALMI for women.

ALMI	β（95%CI）	Detail-P
Mexican American		
<19	0.2 (0.1, 0.2) <0.001	
>19	0.0 (−0.0, 0.0) 0.175	−0.2 (−0.2, −0.1) <0.001
Other Hispanic		
<19	0.1 (0.1, 0.2) <0.001	
>19	0.0 (−0.0, 0.0) 0.624	−0.1 (−0.2, −0.1) <0.001
Non-Hispanic White		
<19	0.2 (0.1, 0.2) <0.001	
>19	−0.0 (−0.0, 0.0) 0.928	−0.2 (−0.2, −0.1) <0.001
Non-Hispanic Black		
<19	0.2 (0.2, 0.2) <0.001	
>19	0.0 (−0.0, 0.0) 0.842	−0.2 (−0.2, −0.2) <0.001
Other Race - Including Multi-Racial		
<19	0.1 (0.1, 0.1) <0.001	
>19	0.0 (−0.0, 0.0) 0.871	−0.1 (−0.1, −0.1) <0.001
Total		
<19	0.2 (0.2, 0.2) <0.001	
>19	0.0 (−0.0, 0.0) 0.516	−0.2 (−0.2, −0.1) <0.001

Table S5. Threshold effect analyses of association between age and HGS for men.

HGS Men	β（95%CI）	Detail-P
Mexican American		
<72	−0.3 (−0.4, −0.3) <0.001	
>72	−1.0 (−1.5, −0.5) <0.001	−0.7 (−1.2, −0.1) 0.017
Other Hispanic		
<72	−0.3 (−0.4, −0.3) <0.001	
>72	−0.8 (−1.4, −0.3) 0.004	−0.5 (−1.1, 0.1) 0.088
Non-Hispanic White		
<72	−0.3 (−0.3, −0.2) <0.001	
>72	−1.0 (−1.2, −0.8) <0.001	−0.7 (−0.9, −0.6) <0.001
Non-Hispanic Black		
<72	−0.3 (−0.4, −0.3) <0.001	
>72	−1.0 (−1.3, −0.6) <0.001	−0.7 (−1.0, −0.3) <0.001
Other Race - Including Multi-Racial		
<72	−0.3 (−0.3, −0.2) <0.001	
>72	−0.9 (−1.3, −0.5) <0.001	−0.6 (−1.1, −0.2) 0.005
Total		
<72	−0.3 (−0.3, −0.3) <0.001	
>72	−1.0 (−1.1, −0.8) <0.001	−0.7 (−0.8, −0.5) <0.001

Table S6. Threshold effect analyses of association between age and HGS for women.

HGS Women	β（95%CI）	Detail-P
Mexican American		
<66	−0.2 (−0.2, −0.1) <0.001	
>66	−0.4 (−0.6, −0.2) <0.001	−0.2 (−0.4, 0.0) 0.084
Other Hispanic		
<66	−0.2 (−0.2, −0.1) <0.001	
>66	−0.5 (−0.6, −0.3) <0.001	−0.3 (−0.5, −0.1) <0.001
Non-Hispanic White		
<66	−0.2 (−0.2, −0.1) <0.001	
>66	−0.5 (−0.5, −0.4) <0.001	−0.3 (−0.4, −0.2) <0.001
Non-Hispanic Black		

<66	−0.2 (−0.2, −0.1) <0.001	
>66	−0.5 (−0.5, −0.4) <0.001	−0.3 (−0.4, −0.2) <0.001
Other Race - Including Multi-Racial		
<66	−0.1 (−0.2, −0.1) <0.001	
>66	−0.4 (−0.6, −0.3) <0.001	−0.3 (−0.5, −0.1) <0.001
Total		
<66	−0.2 (−0.2, −0.2) <0.001	
>66	−0.5 (−0.5, −0.4) <0.001	−0.3 (−0.4, −0.3) <0.001
